# Rich table but short life: Diffuse idiopathic skeletal hyperostosis in Danish astronomer Tycho Brahe (1546-1601) and its possible consequences

**DOI:** 10.1371/journal.pone.0195920

**Published:** 2018-04-19

**Authors:** Sacha Kacki, Petr Velemínský, Niels Lynnerup, Sylva Kaupová, Alizé Lacoste Jeanson, Ctibor Povýšil, Martin Horák, Jan Kučera, Kaare Lund Rasmussen, Jaroslav Podliska, Zdeněk Dragoun, Jiří Smolík, Jens Vellev, Jaroslav Brůžek

**Affiliations:** 1 Department of Archaeology, Durham University, Durham, United Kingdom; 2 PACEA–UMR 5199, University of Bordeaux, Pessac, France; 3 Department of Anthropology, National Museum, Prague, Czech Republic; 4 Laboratory of Biological Anthropology, Institute of Forensic Medicine, University of Copenhagen, Copenhagen, Denmark; 5 Department of Anthropology and Human Genetics, Faculty of Science, Charles University, Prague, Czech Republic; 6 Institute of Pathology, First Faculty of Medicine, Charles University and General University Hospital in Prague, Prague, Czech Republic; 7 Department of Radiology, Na Homolce Hospital, Prague, Czech Republic; 8 Nuclear Physics Institute of Czech Academy of Sciences, Husinec-Řež, Czech Republic; 9 Institute of Physics, Chemistry and Pharmacy, University of Southern Denmark, Odense, Denmark; 10 Department of Archaeology, National Heritage Institute, Prague, Czech Republic; 11 Institute of Chemical Process Fundamentals of Czech Academy of Sciences, Prague, Czech Republic; 12 Department of Culture and Society–Section for Medieval and Renaissance Archaeology, Aarhus University, Højbjerg, Denmark; University of Otago, NEW ZEALAND

## Abstract

The exhumation of Danish astronomer Tycho Brahe (1546–1601) was performed in 2010 to verify speculative views on the cause of his death. Previous analyses of skeletal and hair remains recovered from his grave refuted the presumption that he died from poisoning. These studies also outlined the possibility that he actually died from an acute illness, echoing the rather vague and inaccurate testimony of some historical records. We performed a detailed paleopathological analysis of Tycho Brahe’s skeletal remains, along with a reconstruction of his diet based on carbon and nitrogen stable isotopes analysis and an estimate of his physical status (relative body fat) based on medullar and cortical dimensions of the femoral shaft. The astronomer’s remains exhibit bone changes indicative of diffuse idiopathic skeletal hyperostosis (DISH). The study further allows us to classify him as obese (100% reliability according to our decision tree designed from Danish males), and points out his rich diet (high input of animal protein and/or marine resources) and high social status. Comorbidities of DISH and obesity are reviewed, and their influence on health status is discussed. We further consider some conditions associated with metabolic syndrome as possible causes of Tycho Brahe’s final symptoms (urinary retention, renal failure and coma), including diabetes, alcoholic ketoacidosis and benign prostatic hypertrophy. Although a definite and specific diagnosis cannot be established, our study points to today’s civilization diseases often associated with DISH and metabolic syndrome as the possible cause of death of Tycho Brahe.

## Introduction

Danish astronomer Tycho Brahe (1546–1601) is world renowned for his contribution to the foundation of modern observational astronomy, and also the controversy surrounding the circumstances of his death. He was only 54 years old when he died in Prague, on October 24^th^, 1601, after 11 days of sudden illness. His first symptoms appeared shortly after attending a banquet at the Count of Rosenberg. During this banquet, he had allegedly held his urine longer than was his habit due to etiquette. According to contemporary accounts of his illness, he was thereafter no longer able to urinate. There are three testimonies about the last days of Tycho Brahe–those of Johannes Jessenius, Johannes Kepler, and the young German doctor Johannes Wittich. The first two described symptoms evocative of uremia. Jessenius had reported that “Urine retention and strong pain followed. Bladder inflammation, which—as is usually the case—was immediately accompanied by continuous fever, and from this quite slight delirium arose.” [1] Jessenius’s testimony also mentions that by the 11^th^ day, his delirium had subsided and Brahe regained consciousness, but only for a few hours before he died [2]. Similarly, Kepler wrote that Brahe “endured five days and nights of agony, unable to sleep. Uninterrupted insomnia followed; intestinal fever; and little by little, delirium.” [1,3] Wittich, who was in Prague when Brahe passed away but did not treat the astronomer himself, concluded that a kidney stone was the cause of his anuria [1,3,4]. This third account, which has become the most popular one, claims that Brahe died because of a ruptured bladder [5].

Some alleged that Brahe’s death was not accidental and that the organ failure described in physicians’ accounts were actually the result of poison ingestion. The first of these conspiracy theories was revealed shortly after his death by William Shakespeare in the play Hamlet (1603). More recently, scholars argued that the ruptured bladder could be considered indirect evidence for Hg poisoning, which can result in uremia and kidney failure [6,7]. This assertion has been challenged by other researchers, who argue that bladder rupture is a rare and unlikely occurrence [1,3,4].

The uncertainty around the circumstances of Brahe’s death was one of the reasons why the renaissance astronomer was recently exhumed from his final resting place in the Church of Our Lady before Týn in Prague. The opening of his grave and subsequent study of his remains were performed by a Danish-Czech team under the direction of the National Heritage Institute in Prague on November 15^th^–19^th^, 2010 [8]. This was actually the second opening of his grave; it was previously opened in 1901 by physician and anthropologist Jindrich Matiegka, with the purpose of proving that his remains were still in the grave [9]. At that time, hair samples from the scalp and beard were obtained. A sizeable part of a large red textile present in the tomb was cut into small pieces and given away as mementos of the day. The original coffin, where the astronomer was buried, was broken and the remains placed into a smaller tin coffin, which was then replaced in the grave. The 2010 reopening led to the rediscovery of this container, which still contained most of the bones of the astronomer. The authenticity of his skeletal remains can be considered as very certain, as the tin coffin was still sealed when extracted in 2010; upon opening, the bones completely matched the 1901 description [9], including facial bones with mummified skin and beard remnants still attached. In addition, its authenticity is supported by the anthropological analysis, which demonstrated that the skeleton was with absolute certainty that of a male individual whose age at death was approximately 50 years [10].

The purpose of the Danish–Czech research team was mainly to collect samples of hair, bones, and teeth to determine concentration of Hg and other chemical elements in the remains and to discuss whether or not Brahe had been poisoned. These investigations revealed that Brahe could have been exposed to small doses of Hg in the last weeks of his life, possibly due to administration of his own *Elixir Tychonis*, which contained Hg [5]. He was however not administered potentially lethal doses of Hg, neither shortly before his death nor earlier in his life [11]. Analysis of Brahe’s bones revealed signs of long-term exposure to abnormal levels of Au, whereas hair analysis suggested recent exposure to Fe, As, Ag, Au, and Hg. Possible sources of exposure may have been related to Brahe’s alchemy activities. Concerning Au, exposure to gold cutlery, involvement in gold plating, and use of gold leaf added to wine can also be considered. It is noteworthy that the concentration of Au, Ag, and a few other elements began to decrease 2 months before his death, suggesting a discontinued exposure to these elements [5]. In addition, histomorphometric examination of a sample from the anterior iliac crest was performed and revealed that bone mineralization was within normal limits. This finding excludes the presence of disorders causing abnormal mineralization, such as renal osteodystrophy, nutritional osteomalacia, vitamin D and calcium deficiencies from gastrointestinal malabsorption syndromes, intoxication with certain metals, and phosphate deficiency [5].

The results of the previous studies thus ruled out the hypothesis that Brahe was poisoned with Hg; but they do not to reveal the precise circumstances of his death. More generally, nothing is known about Brahe’s health during the last years of his life, nor shortly before his death, with the exception of the aforementioned final 11 days [1]. It is noteworthy, however, that previous studies investigated these questions through chemical analysis and histomorphometric examination only, while pathological bone changes that were present on his skeleton were overlooked as a source of potential information. Preliminary paleopathological evaluation showed that Brahe’s remains exhibited extensive remodeling in all spinal segments, as well as changes at the ligament and muscle insertions [12]. The etiology of these skeletal changes was, however, not assessed further. The aim of the newly undertaken investigations was therefore to assess Brahe’s physical condition and health, based on a multi-proxy, disease-focused approach of his skeletal remains.

In this paper, we present the result of the first comprehensive paleopathological analysis of Tycho Brahe’s skeletal remains, providing evidence that the aforementioned bones changes were caused by diffuse idiopathic skeletal hyperostosis (DISH). We further discuss risk factors for the condition related to Brahe’s lifestyle (i.e. dietary excesses and obesity), as revealed by reconstruction of his diet based on carbon and nitrogen stable isotopes analysis and an estimate of his physical status (relative body fat) based on medullar and cortical dimensions of his femoral shaft. We also offer a plausible clarification of the circumstances of his death based on new data on his physical condition and health. Although DISH is nowadays a relatively common condition in older males and has been previously reported in archaeological material, the current study is one of the rare to provide multidisciplinary evidence of the possible causes and consequences of this condition, which still deserves more attention in both the medical sciences and paleopathology with respect to its etiology and life-threatening comorbidities. The study of Brahe’s remains furthermore contributes to the osteobiography of this famous person, who in many ways was an example of a true “Renaissance” man of nobility.

## Material and methods

**Fig 1:**
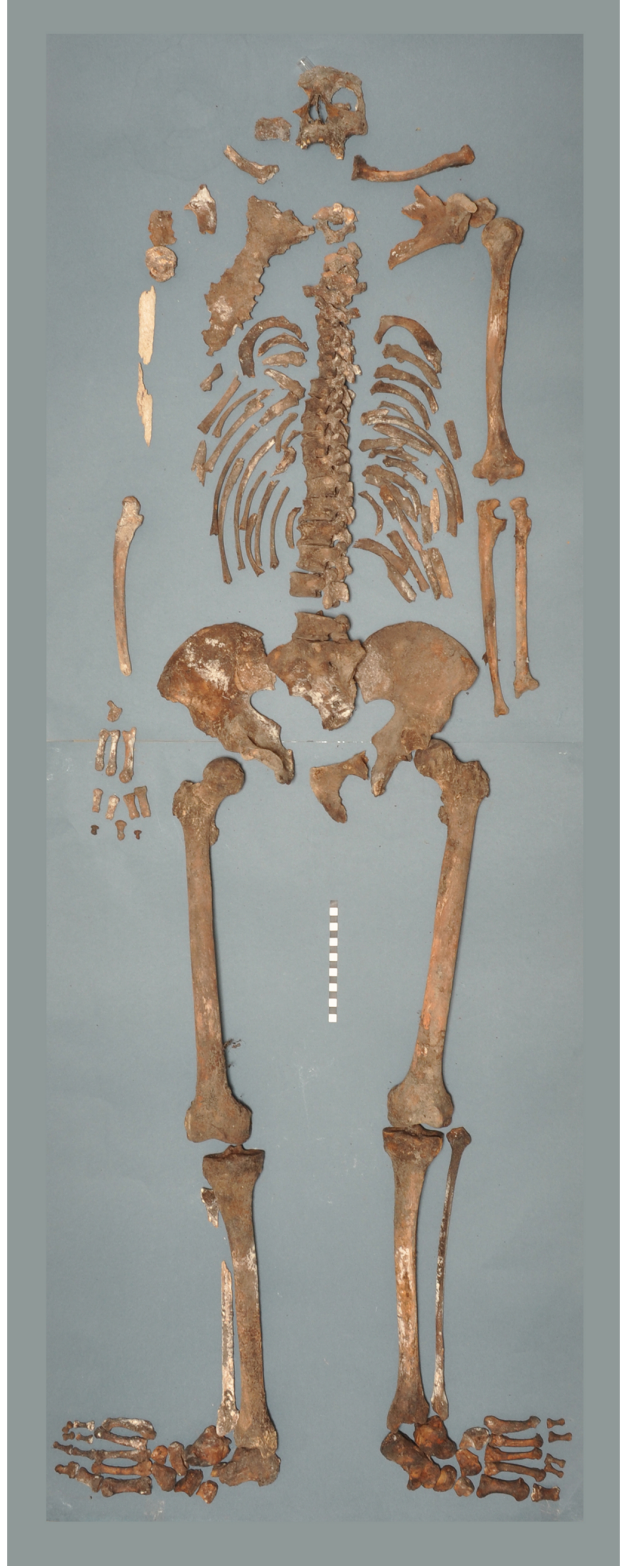
The skeletal remains of Tycho Brahe (Photo Marek Jantač).

The bones recovered from the coffin are those of a single and almost complete adult skeleton (Fig 1). A few bone elements are missing, probably due to loss or removal at the time of the 1901 reopening of the grave. The skull is only represented by the facial bones and the lower part of the frontal bone. The mandible is missing, as well as most of the cervical vertebrae (C1, C3–C5). With regards to the post-cranial skeleton, the right radius and bones of the left hand are missing, as well as most intermediate and distal phalanges of the right hand and both feet. The other skeletal elements are present and generally well preserved, with the exception of the bones of the right upper limb that have suffered taphonomic breakage and cortical erosion.

Degenerative changes of the spine (spondylosis), osteoarthritic changes at extraspinal sites, and entheseal changes were recorded and described according standard scoring systems developed for paleopathology [13–16]. Macroscopic examination was supplemented by X-ray of each preserved bone, performed at the Department of Anthropology, National Museum in Prague. In addition, all bones were scanned by computed tomography (CT) using a Somatom Sensation 16 (Siemens, Erlangen, Germany) with a slice thickness of 0.4 mm, 0.75 mm increment. CT examination was performed at the Department of Radiology, Hospital Na Homolce in Prague. Surface models were segmented using TIVMI software (http://projets.pacea.u-bordeaux.fr/TIVMI/)).

### Paleopathological assessment of DISH

DISH is a common but often unrecognized systemic disorder characterized by ossification adjacent to the anterior longitudinal ligament in the spinal column and ossification of extraspinal entheses and ligaments [17–19]. Various diagnostic criteria for DISH have been published in the clinical and paleopathological literature (Table 1). Most of them focus on spinal changes and the number of vertebrae that have to be affected, however variable according to the methods. Some authors proposed that the diagnosis of DISH is restricted to the case where calcification and ossification at the site of the anterior longitudinal ligament leads to the complete fusion of the vertebral bodies [20–22]. However, close inspection of cadavers of individuals radiographically diagnosed with DISH reveal areas in which true ankylosis is not present, but rather, irregular osseous outgrowths interdigitate, their undulating surface being separated by thin layers of fibrous tissue [23,24]. Consequently, some authors proposed to consider any flowing ossification confined to the right side of the thoracic vertebrae, with or without fusion, as a diagnostic feature of DISH [17,25,26]. Cases where bony bridges are incomplete have been termed early-stage DISH by some authors [22,27], although to our knowledge no study has demonstrated the relation between degree of bone fusion and disease duration. One of the key features of the spinal manifestations of DISH is that they usually involve only the right side of the thoracic vertebrae, whereas in the lumbar spine both the right and left sides of the vertebral body may be equally involved [23,28]. The unilaterality of changes in the thoracic spine is thought to be due to the left sided descending aorta, whose pulsation may inhibit bone production [29,30].

**Table 1:** Diagnostic criteria for diffuse idiopathic hyperostosis in the clinical and paleopathological literature (modified and completed after [31]).

Criteria	Spinal changes	Extra-spinal changes	Exclusion criteria
Bywaters et al. 1966 [32]	Complete bridge between two vertebral bodies (any level)	Not required	Signs of degenerative disc disease
Julkunen 1971 [20]	Two complete bridges between contiguous or non-contiguous vertebral bodies (thoracic region)	Not required	None
Harris 1974 [33]	Hypertrophic spurs with at least two bony bridges (thoracic region)	Not required	Sacroiliitis; changes evocative of other spinal diseases
Resnick and Niwayama 1976 [17]	Flowing calcification and ossification along the anterolateral aspect of four contiguous vertebral bodies (any level); with or without ankyloses	Not required	Intervertebral disc space narrowing; signs of extensive degenerative disc disease; apophyseal joint ankylosis; sacroiliac joint erosion, sclerosis, or intraarticular osseous fusion
Arlet and Mazières 1985 [34]	Flowing ossification along the anterolateral aspect of three contiguous vertebral bodies (lower thoracic region); with or without ankyloses	Not required	Intervertebral disc space narrowing; signs of extensive degenerative disc disease; apophyseal joint ankylosis; sacroiliac joint erosion, sclerosis, or intraarticular osseous fusion
Utsinger 1985 [21]			
• Definite DISH	Bridging of four contiguous vertebral bodies (thoracolumbar region)	Not required	Signs of degenerative disc disease; apophyseal joint ankylosis
• Probable DISH	Bridging of two vertebral bodies	Bilateral patellar tufting, heel spurring and olecranon tufting	
• Possible DISH	Bridging of two vertebral bodies	Not required	
	Not required	Symmetrical enthesophytes	
Crubézy and Crubézy-Ibáñez 1993 [35]			
• Definite DISH	Bridging of three vertebral bodies (lower thoracic region) / four vertebral bodies (any level)	Not required	Sacroiliac joint erosion, sclerosis, or intraarticular osseous fusion
• Probable DISH	Bridging of two vertebral bodies	Symmetrical enthesopathies (posterior calcanei, olecranons, and upper patellae)	
• Possible DISH	Bridging of two vertebral bodies	Not required	
	Not required	Symmetrical enthesopathies	
Rogers and Waldron 2001 [26]	Hyperostosis affecting three vertebral bodies (thoracic region); with or without ankylosis	Calcification or ossification in ligaments and entheses	None
Kacki and Villotte 2006 [25]			
• Definite DISH	Flowing ossification along the anterolateral aspect of four contiguous vertebral bodies (thoracic region); with or without ankyloses	Not required	Intervertebral disc space narrowing; apophyseal joint ankylosis
• Probable DISH	Flowing ossification along the anterolateral aspect of two contiguous vertebral bodies (thoracic region); with or without ankyloses	Symmetrical entheseal changes (patellar tufting, heel spurring, olecranon tufting)	
Waldron 2008 [22]	Fusion of four contiguous vertebrae (thoracic region)	Ossification into entheses and ligaments	None

Besides vertebral changes, diagnostic criteria for DISH may also include calcification or ossification in the extraspinal ligaments and entheses [21,25,26,35]. Entheseal changes and ligament ossification in DISH patients are reported to be particularly common in the pelvis (iliac crest, ischial tuberosity, sacrotuberous, and iliolumbar ligaments), trochanters, calcaneus (Achilles tendon and plantar aponeurosis) and olecranon (triceps brachii) [23,26,36,37]. Moreover, DISH may result in the formation of para-articular osteophytoses in the sacroiliac joint that can extend from its margin and even bridge the articulation [23]. A previous paleopathological study showed a significant association between sacroiliac fusion by bridging osteophytes and the presence of DISH [38], so it can be considered as an additional diagnostic feature of the condition.

In the current study, DISH was diagnosed relying primarily on the widely used diagnostic criteria of Resnick and Niwayama [17]. This method is favored since it is one of the most restrictive, both in terms of the number of affected vertebrae and with regards to exclusion criteria. The latter prevents a wrongful diagnosis. Symmetrical entheseal changes at extraspinal sites and bridging para-articular osteophytoses in the sacroiliac joint, although not taken as primary criteria in the diagnosis, were considered as additional evidence of the condition.

### Stable isotope analysis

For stable isotope analysis, 300 mg of compact bone was sampled from the femoral shaft of Tycho Brahe. For comparative purposes, we also analyzed a similar sample collected from the skeleton of Brahe’s wife, Kirsten Jørgensdatter, whose skeletal remains were obtained during the same exhumation and whose identity was already confirmed by Matiegka [9]. Femurs were preferred for sampling instead of more routinely used bones (i.e. ribs) because skeletal remains of Kirsten Jørgensdatter were found in such a state, that we could not be sure that present rib fragments belonged to her. Due to the distinct turnover rate of different parts of the skeleton [39], the same bone element was needed for sampling to capture the dietary signal of similar life period of both individuals [40].

Collagen extraction proceeded according to the Longin [41] method, modified by Bocherens [42]. Elemental analyses were performed using a Europa Scientific EA elemental analyzer connected to a Europa Scientific 20–20 IRMS for carbon and nitrogen isotope analysis at Iso-Analytical Limited, Crewe (UK). The uncertainty of isotopic measurements calculated on different standard replicates (IA-R042, IA-R038, IA-R046 and IA-R05, IAR06) was less than 0.1‰ (1 SD) for both nitrogen and carbon.

Due to the slow bone turnover rate in the femoral shaft, isotopic values from analyzed samples represent the average dietary signal for a vast period of time, spanning from adolescence through adulthood [43]. According to his biographers, Brahe spent the majority of his adult life in Denmark, arriving in Prague only 2 years before his death when becoming Royal Mathematician at the court-in-exile of the Holy Roman Emperor, Rudolf II; his wife spent 5 years in Prague. Thus, to characterize the dietary behavior of the two individuals, we used comparative datasets for northwestern European contexts. Human and animal isotopic data were taken from datasets from late medieval Denmark [44–46], early modern Belgium [47], and 18^th^ to 20^th^ centuries Copenhagen [48]. An early modern population sample from Prague (15^th^ to 16^th^ centuries merchants and 18^th^ century soldiers [49]) was also included for comparison. The collagen extraction methods applied in these studies differed in several aspect including acid concentrations and duration of demineralization, the demineralization of bone chunks vs. powders, the use of NaOH or ultra-filtration. None of these factors, however, have been shown to have a significant impact on carbon and nitrogen isotopic values of bone collagen in experimental studies [50–53]. Thus, the data are considered to be well comparable.

### Physical status estimation based on relative body fat

Researches demonstrated that the methods classically used to estimate body mass from the skeleton are not reliable for estimating individual body mass, nor they are reliable for estimating physical status or body size (i.e. emaciation, physiological or normal body mass, obesity) [54–56]. This is mostly because the data used to create the regressions for estimating body mass consist of average or estimated body masses for population samples or self-recalled body mass at 18 years of age [57–61]; and because the skeletal variables used do not change much after the end of the skeletal maturation and are thus not able to track potential changes in physical status during adulthood [57,61].

However, the femoral diaphysis is more labile and functionally adapts and remodels according to the biomechanical constrains imposed upon it [57,62,63]. Therefore, we built a decision tree based upon femoral bone data from a Danish sample of 36 males with known mass and body composition to estimate Brahe’s physical status. The sample is part of a randomly constituted dataset of whole-body computed tomography (CT) scans performed on cadavers at the University of Copenhagen, Department of Forensic Medicine, Unit of Forensic Anthropology in Copenhagen, Denmark (described in [64]). No formal ethical consent is needed from Danish ethical committees to work with CT images of deceased humans. Autopsies are mandated by the police and CT scans are part of the routine investigation at the University of Copenhagen, Department of Forensic Medicine. The Department of Forensic Medicine adheres to Danish accreditation standards regarding data security. All personal data are removed from the images; only age, sex, weight and height data were retained. Bodies were scanned within 3 days after death and exhibited very limited or no sign of decomposition. Age was recorded and bodies were weighed on an electronic scale with clothes removed prior to autopsy.

Body mass index is a common way of measuring physical status, but it does not measure fatness, and can therefore only serve as an approximate indication of obesity or emaciation, which are the result of an excess or a defect, respectively, of adipose tissue [65]. Therefore, we chose to use a more conservative definition of obesity based on body composition. At the tissue level, the body can be divided between fat mass (FM) and fat-free mass (FFM, i.e. the sum of muscles, organs and bone). FM and FFM were derived from the assessment of adipose, lean and skeletal tissue volumes on CT scans and known density of the founder (0.92 kg per liter), from which derived mass was subtracted from known weight (described in [64]). Body fat proportion (BF%) was calculated as FM/(FM+FFM)×100. Obesity is defined at the threshold of greater than or equal to 25 BF% for males [65]. The sample is composed of eight individuals of normal weight and 28 who were considered obese.

Decision trees were generated from the classification and regression tree (CART algorithm) [66]. Decision trees are devices used by investigators to determine the most discriminant variables with regard to categorical variables. Essentially, decision trees indicate which bone variables are decisive for classifying individuals into normal or obese categories. The number of terminal leaves in the decision trees is determined by minimizing the error of prediction using a leave-one-out cross-validation.

Only the left femora were used for analysis. To check for the assumption regarding the limited power of femoral external dimensions for estimating physical status, we measured the neck breadth, head supero-inferior breadth, head antero-posterior breadth, bicondylar breadth, and included all those dimensions into the variables that the tree could select for distinguishing between obese and normal categories. Additionally, precise data of the femoral diaphyseal bone were extracted using custom-built software that automatically reslices the bone to align it along the medial axis [67]: cortical, medullary, and total areas of cross-sections located at 20%, 35%, 50%, 65% and 80% of the femoral maximum length were recorded, as well as medio-lateral (ML) and antero-posterior subperiosteal and medullary diameters of the same cross-sections. All data were adjusted by maximum femoral length to minimize the effect of stature variation on the results. The most discriminant skeletal variables identified by the tree for classifying individuals between categories of physical status were then measured on a CT scan of Brahe’s left femur, from which data were extracted following the same procedure.

## Results

### Description and diagnosis of pathological changes

The preserved skeletal remains show several pathological lesions, primarily concentrated in the vertebral column and, to a lesser extent, in the peripheral entheses (S1, S2 and S3 Tables). A detailed description of each of the pathological changes is provided below.

**Fig 2:**
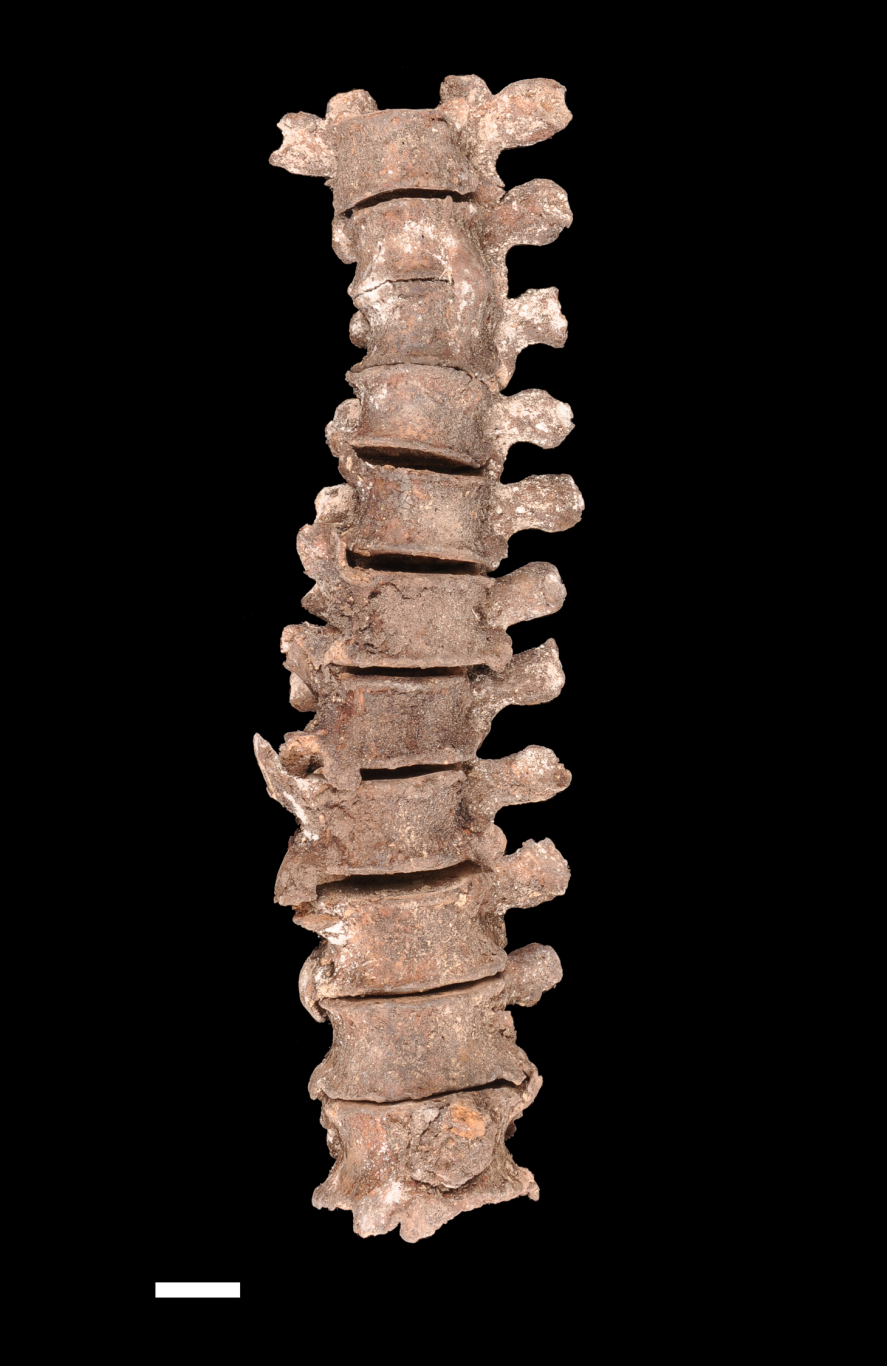
Anterior view of the thoracic spine (T2–T12). A flowing ossification is present along the right side of the vertebrae. Also note the left sided bony bridge that leads to fusion of T3 and T4. Scale: 2 cm (Photo Marek Jantač).

**Fig 3:**
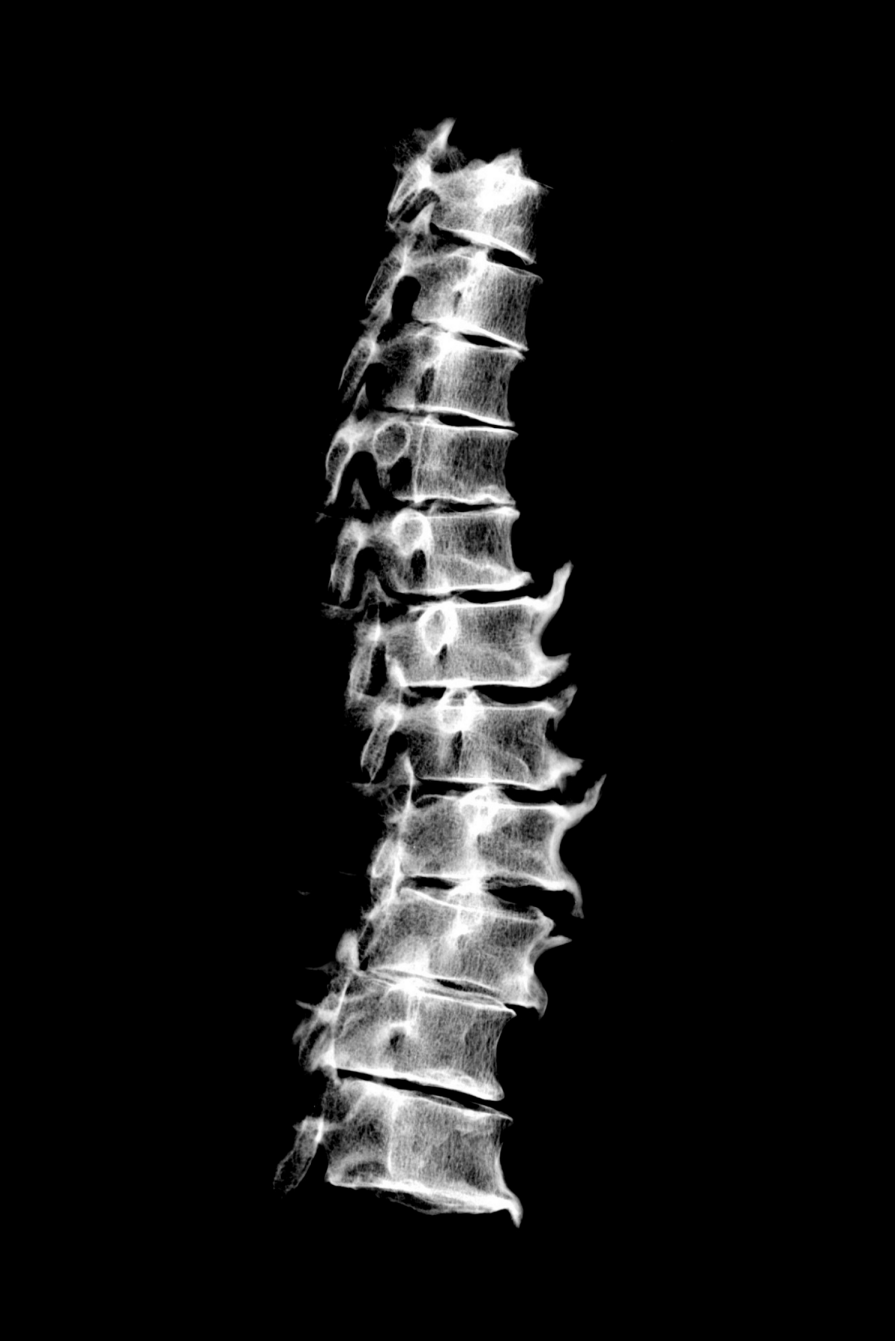
Radiograph of the T2–T12 thoracic vertebrae (*norma lateralis*). Note the ossification at the site of the anterior longitudinal ligament that coats the vertebral bodies.

The cervical spine did not show pathological changes, except from slight osteophytosis on the anterior border of the lower plate of C7. On the contrary, the thoracic vertebrae exhibited major pathological changes in the form of massive bone formation on the vertebral bodies. All but the T1 and T2 vertebrae were affected. T3 and T4 were fused together by a bony bridge located on the left side and a forming bony bridge was present on the contralateral side. The section of the spine situated below (i.e. T5–T10) exhibited flowing ossifications restricted to the right side of the vertebral bodies, at the site of the anterior longitudinal ligament (Fig 2). These dense bony outgrowths projecting upward or downward were unfused but in contact and interdigitating. T11 and T12 were less severely affected, showing small bridging enthesophytes on both their right and left aspects. X-ray examination of the thoracic spine revealed that the bone formations had a dense cortex and coated the vertebral bodies without destruction of the normal, underlying cortical bone (Fig 3). No obvious changes in the trabecular organization of the vertebral bodies were apparent. Similar candle-flame-shaped ossifications were present bilaterally on the L1 and L2 vertebrae, extending down to L3 on the left side. The last two lumbar vertebrae and the sacrum were unaffected by any pathologic lesion. Along the spine, the intervertebral spaces appeared to be preserved and no pathological changes were observed in the apophyseal joints either on macroscopic and radiologic examination.

Both hip bones showed some degree of hyperostosis at the attachment sites of ligaments, particularly those of the inguinal ligament (anterior superior iliac spine), iliolumbar ligament (posterior part of the inner lip of the iliac crest), iliofemoral ligament (anterior inferior iliac spine), and sacrotuberous ligament (medial margin of the ischial tuberosity). Marked exostoses were present on the posterior aspect of the iliac crests and both ischial tuberosities are grossly remodeled, showing an irregular surface with osseous production and foramina. On the right hip bone, small bony spurs had developed from the border of the superior and inferior pubic rami, projecting in the obturator foramen.

**Fig 4:**
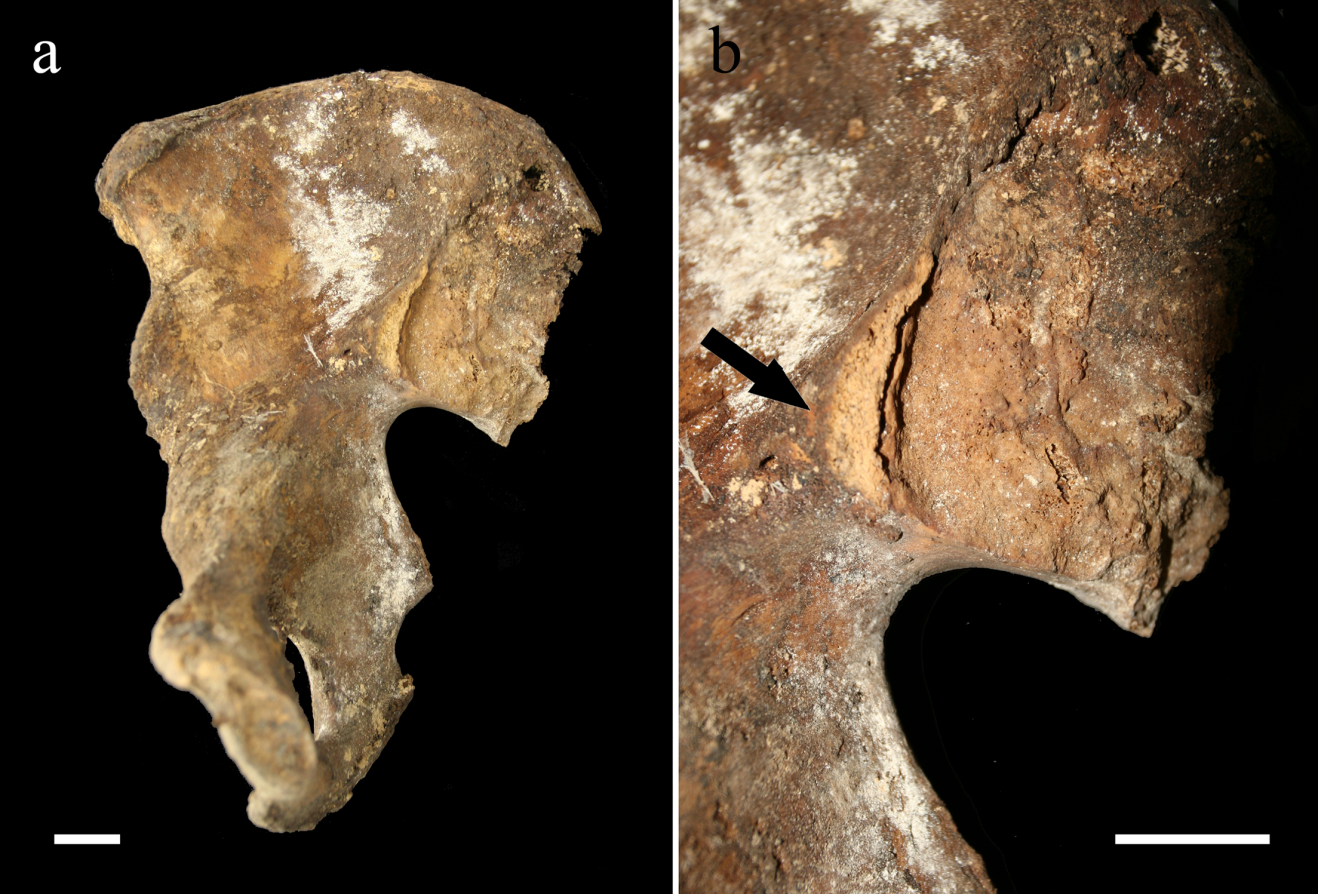
**The right innominate (a) and close-up view of its sacroiliac region (b).** The pictures show the broken bony bridge (arrow) that connected the hip bone to the sacrum. Scale: 2 cm (Photo Marek Jantač).

The right innominate also showed thick, smooth exostosis lying at the apex of the auricular surface (Fig 4). This bony outgrowth had been broken post-mortem but initially united the hip bone with the sacrum, as demonstrated by fitting surfaces with the remaining bone bridge at the sacrum. The sacroiliac fusion resulted from sacral ligaments ossification only, without intra-articular involvement. The synovial part of the auricular surface showed no pathological changes except from surface remodeling due to aging, similar to what was observed at the left hip bone.

Only a few pathological changes were present in the upper limb bones. The sternal ends of both clavicles showed articular lesions evocative of osteoarthritis, i.e. marginal lipping (osteophytosis) and pitting on the joint surface. Slight osteophytosis was also present on the inferior margin of the left glenoid fossa. Apart from that, the only other bone modification observed was a large bony spur at the attachment site of the triceps brachii muscle on the left ulna. The contralateral bone was heavily damaged, making it impossible to assess whether or not it was also affected.

**Fig 5:**
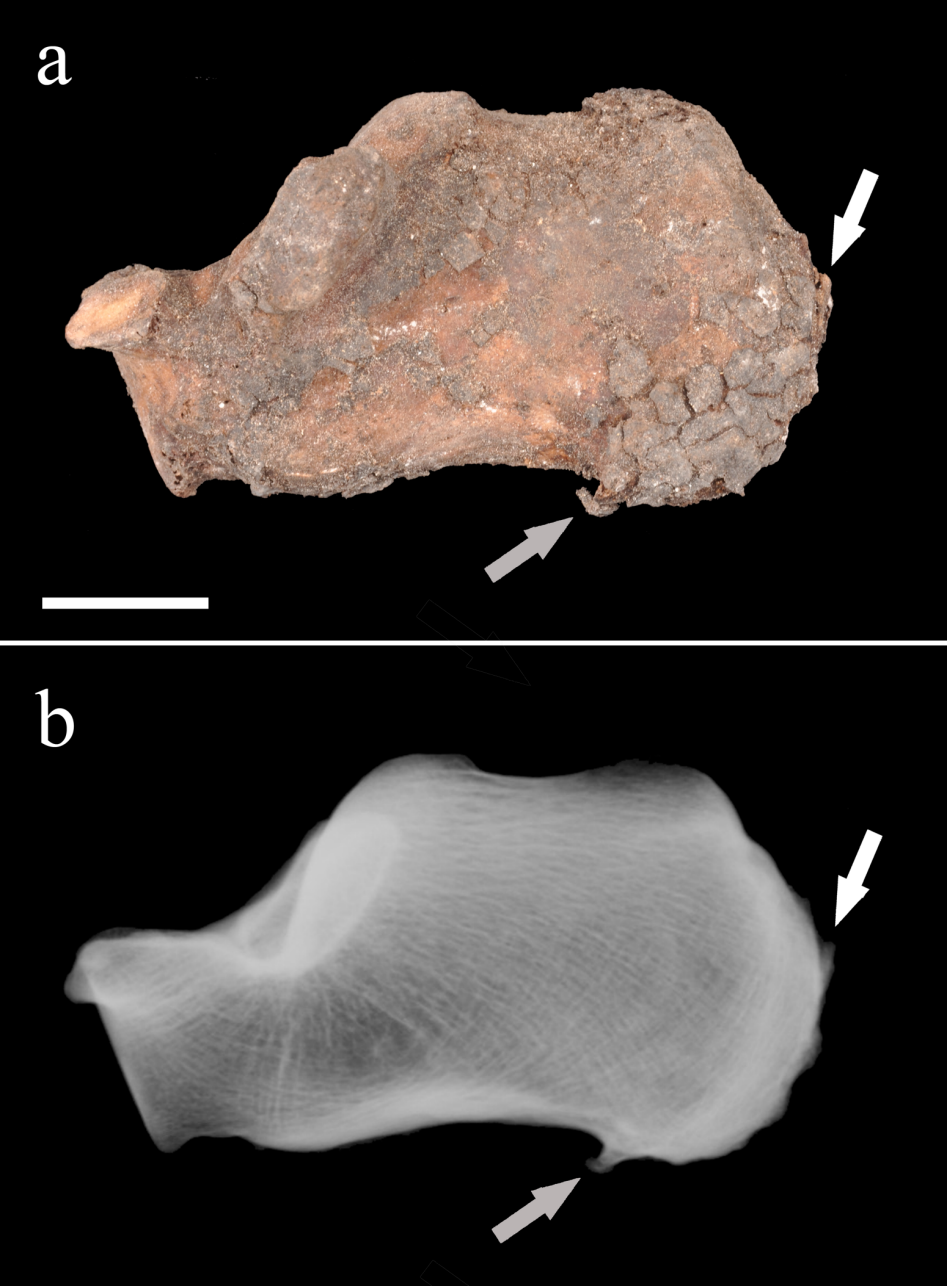
**Macroscopic (a) and radiologic (b) aspects of the right calcaneus (medial view).** Note the presence of the enthesophyte at the insertion of the triceps sural muscle (white arrows) and the heel spur on the plantar surface (grey arrows). Scale: 2 cm (Photo Marek Jantač).

Entheseal changes were present bilaterally in the lower limb bones, especially in the femora, tibiae, and calcanei. The femora showed enthesophytes on the greater and lesser trochanters (attachment sites of the gluteus minimus and gluteus medius, and of the iliopsoas muscle, respectively), as well as bony spurs in the trochanteric fossa (insertion of the external obturator muscle) and formation of an irregular bony crest at the attachment site of the gluteus maximus muscle on the posterior shaft. A large bony crest was also apparent in the posterior aspect of both tibiae at the insertion of the soleus muscle. The right and left calcanei exhibited enthesophytes at the insertion of the Achilles tendon, as well as heel spurs that developed on the plantar aspect of the bones (Fig 5). The other foot bones (tarsal, metatarsal, and phalanges) only showed slight osteoarthritic changes.

Systematic X-ray examination of the bones did not reveal pathological changes, other than those identified through macroscopic examination.

Although hyperostotic lesions of the spine and the appendicular skeleton of Tycho Brahe may have resulted from various causes, in the current case their distribution throughout the skeleton and their anatomic features ruled out most of the possible diagnosis. Degenerative processes alone appear as an unlikely cause of recorded bone changes, notably those of the spine. In degenerative joint disease, osteophytes arise from the margins of the vertebral bodies and are horizontally oriented [23,68,69], in contrast to the massive, vertical bony outgrowths recorded here, which encroach on the lateral borders of the vertebral bodies. Moreover, bone fusions, such as those affecting the T3–T4 vertebrae and the right sacroiliac joint are an uncommon feature in degenerative joint disease, except in very old individuals [23,70]. The spondyloarthropathies (SpA), a group of inflammatory rheumatic disorders that share common genetic predisposing factors and clinical features, may cause vertebral and sacroiliac fusion, as well as extraspinal enthesitis [23,71–73]. However, these diseases generally involve both the vertebral bodies and the apophyseal joints, and the intervertebral space is not preserved [23,74]. Furthermore, in SpA sacroiliac ankylosis is due to synovial inflammation resulting in intra-articular destruction and fusion [75,76] and the entheseal changes are mainly osteolytic [77,78]. Here, the absence of zygapophyseal lesions, signs of sacroiliitis, or erosive lesions of entheses dismissed SpA as a possible diagnosis. Other rare causes of osseous outgrowths of the spine such as acromegaly, hypoparathyroidism, fluorosis, ochronosis, and sternoclavicular hyperostosis can also be ruled out, in the absence of evocative signs of those diseases elsewhere in the skeleton.

DISH is conversely a likely diagnosis of bone changes with regards to their anatomic features and their distribution in the skeleton. The presence of interdigitated exostoses in six adjacent thoracic vertebrae (not counting the T3–T4 ankylosis) in the absence of apophyseal joint ankyloses, sacroiliitis, and signs of degenerative disc disease fulfills Resnick and Niwayama’s diagnostic criteria for the disease [17]. The diagnosis is reinforced by the right sided predominance of the flowing ossification in the thoracic spine. In this respect, it is noteworthy that the bone bridge uniting the left lateral aspect of T3 and T4 is located superior to the aorta, which runs from the lower border of T4 to the lower border of T12. The diagnosis of DISH in Brahe’s skeletal remains is also supported by the presence of symmetrical distributed ossification in entheses and ligaments that are reported to be frequently affected in patients with the condition, i.e. lesions in the pelvis (iliac crest, ischial tuberosity, sacrotuberous, and iliolumbar ligaments), trochanters, calcaneus (Achilles tendon and plantar aponeurosis), and olecranon (triceps brachii). Considering these extraspinal changes along with the spinal ones, the diagnosis of DISH is confirmed using most sets of criteria used for paleopathological diagnosis of the disease (Table 1). Finally, the presence of sacroiliac ankylosis by a thick, smooth exostosis bridging between the apex of auricular surface of the ilium and the upper aspect of the sacral wing is also consistent with the diagnosis of DISH [23,38].

### Reconstruction of diet from stable isotope analysis

For Tycho Brahe, the δ^13^C was -19.0‰ and δ^15^N was 14.7‰. For his wife Kirsten, δ^13^C was −19.4‰ and δ^15^N was 13.4‰. Due to the slow bone turnover rate in the femoral shaft, it is improbable that Tycho Brahe’s isotopic values could be influenced significantly by any of the pathologicalc conditions he suffered during the final period of his life [79]. Moreover, a study has shown that DISH is not among the pathological conditions that have an effect on the isotopic composition of bones [47]. There is no evidence that Kirsten suffered from a long-term chronic disease that could have influenced the results. The numeric values obtained can thus be considered as reliable and interpreted in terms of dietary habits.

**Fig 6:**
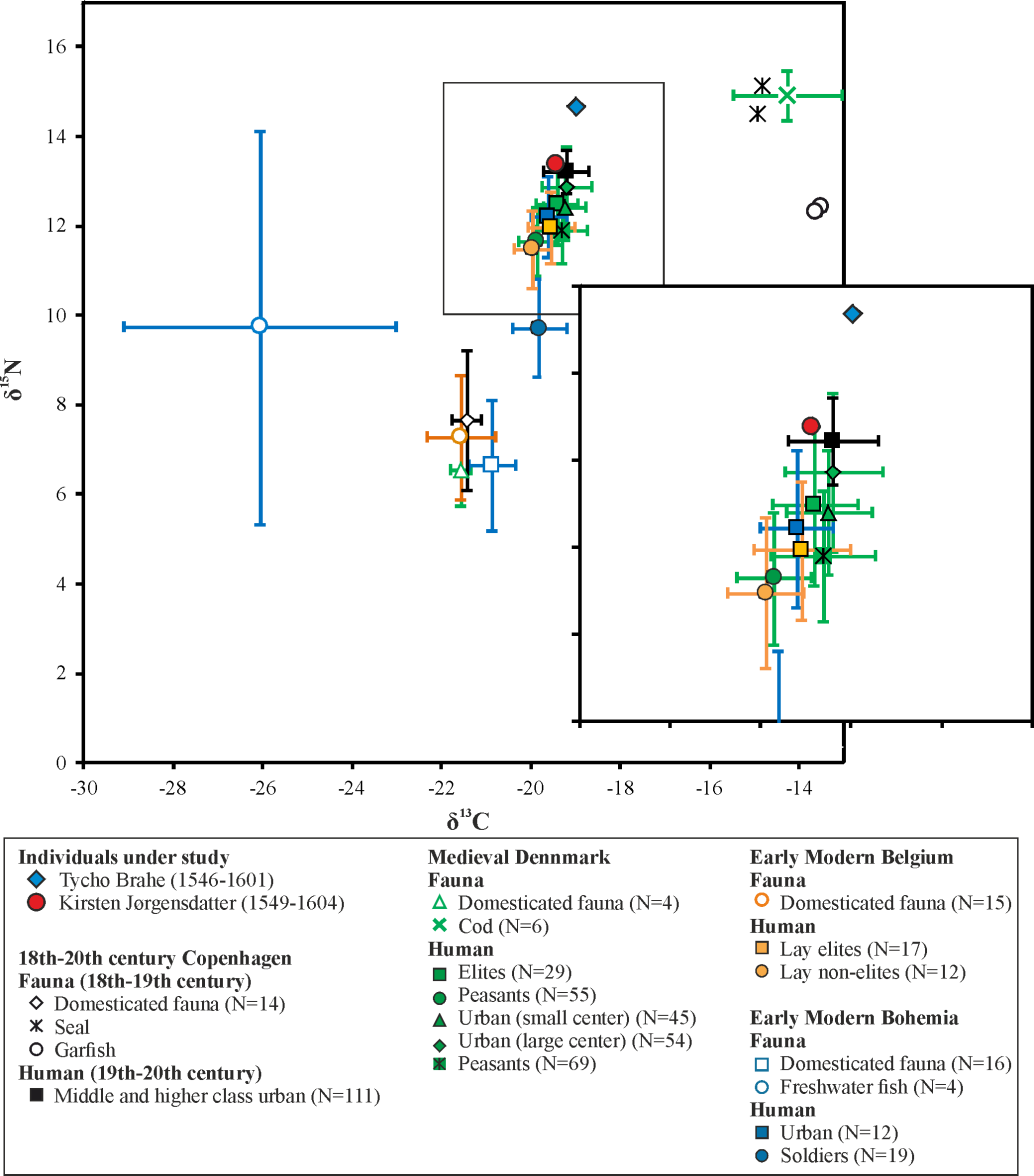
δ^13^C and δ^15^N values in Tycho Brahe and his wife, compared with isotopic datasets from medieval and post-medieval Europe. Isotopic values used for comparative purpose are for faunal and human remains from late medieval Denmark [44–46], early modern Belgium [47], 18^th^–20^th^ centuries Copenhagen [48], and early modern Prague [49,82]. Detailed view at human data is provided on the right. As domesticated fauna, the mean values of the main consumed domesticated species (cattle, sheep/goat, pig) were used.

Animal data taken from comparison datasets suggest some variation in carbon isotopic values according to geographic areas, with the Prague sample being slightly enriched in δ^13^C compared to northwestern European contexts (Fig 6). Nitrogen isotopic values were similar between both contexts (with the exception of the modern Copenhagen sample, which shows slightly higher values), which makes them comparable, at least in terms of animal protein consumption. The human data of northwestern European medieval and early modern populations are usually interpreted as a reflection of a terrestrial based diet with a measurable input of marine protein [45–47]. However, much higher carbon isotopic values were observed in Danish populations from previous periods, who substantially relied on marine protein [80,81], so freshwater or anadromous origin of at least a part of consumed aquatic protein cannot be excluded [44,48]. Interestingly, Prague merchants show similar isotopic values [49], though the lower human-faunal carbon isotopic offsets make the freshwater origin of the aquatic protein more probable. The socioeconomic gradient in diet has been commonly observed, showing a higher amount of animal and/or aquatic protein in the diet of elites and urban populations. This is usually explained by the higher pressure on elite households to follow fasting prescriptions. Thus, the consumption of fish appears to be a marker of the pertinence to the upper class in the late medieval and early modern period [44–49].

In this light, the diet of both sampled individuals has a rather prestigious character with a high input of animal protein and/or aquatic resources (Fig 6). Their skeletal δ^13^C are greater than 2‰ above the animal baseline. As the trophic level shift for carbon is around 1‰, this indicates a marine fish component in their diet [83]. However, as stated above, a freshwater component of the diet cannot be excluded. While Kirsten Jørgensdatter’s nitrogen isotopic value and human-faunal offset is was located within the mean + 1 SD range of the late medieval center of Ribe [45] and 19^th^–20^th^ century Copenhagen [48], Tycho Brahe’s δ^15^N and human-faunal offset is markedly above this range (Table 2). In fact, his nitrogen isotopic value is located at or even above the upper range of the observed variability of all studied datasets. The isotopic values of both individuals are comparable to those of the highest European elites and royal families of that time [84–86]. The isotopic difference between both individuals (0.5 for δ^13^C and 1.3 for δ^15^N) is slightly higher than in other identified couples [84,85], suggesting a higher input of animal (probably marine) protein into Tycho Brahe’s diet.

**Table 2:** Bone collagen δ^13^C and δ^15^N and human-faunal offsets of the studied individuals and comparative datasets (mean ± 1 SD).

Individual/region	Time period	Context	N	Bone sampled	δ^13^C	δ^15^N	Δ^13^C _human-fauna_^a^	Δ^15^N _human-fauna_^a^	Reference
Tycho Brahe	1546–1601	–	–	femur	−19.0	14.7	2.6^b^	8.2^b^	This study
Kirsten Jørgensdatter	1549–1604	–	–	femur	−19.4	13.4	2.2^b^	6.9^b^	This study
Denmark	12^th^–16^th^ century AD	elites	29	tibia, femur, humerus	–19.4 ± 0.5	12.5± 0.9	2.2	6.0	[46]
Denmark	12^th^–16^th^ century AD	peasants	55	tibia, femur, humerus	–19.9 ± 0.4	11.6± 0.8	1.7	5.1	[46]
Denmark	12^th^–16^th^ century AD	urban (small center)	45	tibia, femur, humerus	-19.2±0.4	12.4±0.7	2.4	5,9	[45]
Denmark	13^th^–15^th^ century AD	urban (large center)	54	tibia, femur, humerus	–19.2 ± 0.5	12.9± 0.9	2.4	6.4	[45]
Denmark	12^th^–16^th^ century AD	peasants	69	rib	−19.3 ± 0.6	11.9± 0.7	2.3^b^	5.4^b^	[44]
Czech lands	15^th^–16^th^ century AD	urban	12	femur	−19.6 ± 0.4	12.2± 0.9	1.3^c^	5.6^c^	[49]
Czech lands	18^th^ century AD	soldiers	19	femur	−19.8 ± 0.6	9.7 ± 1.1	1.1	3.1	[49]
Belgium	16^th^–18^th^ century AD	elites	17	rib, long bones	−19.5 ± 0.5	12.0± 0.8	2.1	4.7	[47]
Belgium	16th–18th century AD	non-elites	12	rib, long bones	−19.9 ± 0.4	11.5± 0.9	1.7	4.2	[47]
Denmark	19^th^–20^th^ century AD	urban middle and higher classes	111	phalanx (hand or foot)	−19.2 ± 0.5	13.2± 0.5	2.3	5.6	[48]
Spain	14^th^ century AD	royal family	4	rib, metatarsal	–18.6 ± 0.4	12.5± 0.6	0.6–2.6^d^	6.1–7.9^d^	[85]
England	15^th^ century AD	Richard III	1	rib	−18.7	14.9	2.6	7.7	[86]

^a^ As faunal data, the mean values of the main consumed domesticated species from the corresponding animal sample (cattle, sheep/goat, pig) were used.

^b^ Animal data published by Yoder [46] were used for comparison.

^c^ Animal data of the Czech medieval sample published by Frolík and Kaupová [82] were used for comparison.

^d^ Jiménez-Brobeil et al. [85] published only the mean data for particular species, due to the absence of original data, the presented interval indicates differences between species.

Notably, before coming to Prague, Brahe had his manor and astronomic observatory on the island of Hven, between Denmark and Sweden, a place where marine foodstuffs were probably abundant. As a member of the elite classes, he could have access to the broad spectra of fish, including more expensive freshwater species such as pike [87,88]. Consumption of such fish, as well other delicacies of the privileged people including high quality meat of young animals (e.g. lamb, veal) [89] or wildfowl (e.g. swans, herons, pheasants), could have contributed to the observed high δ^15^N of both individuals. However, it is not possible to distinguish the contribution of these dietary items from present isotopic values.

### Estimation of physical status from the femoral diaphysis

**Fig 7:**
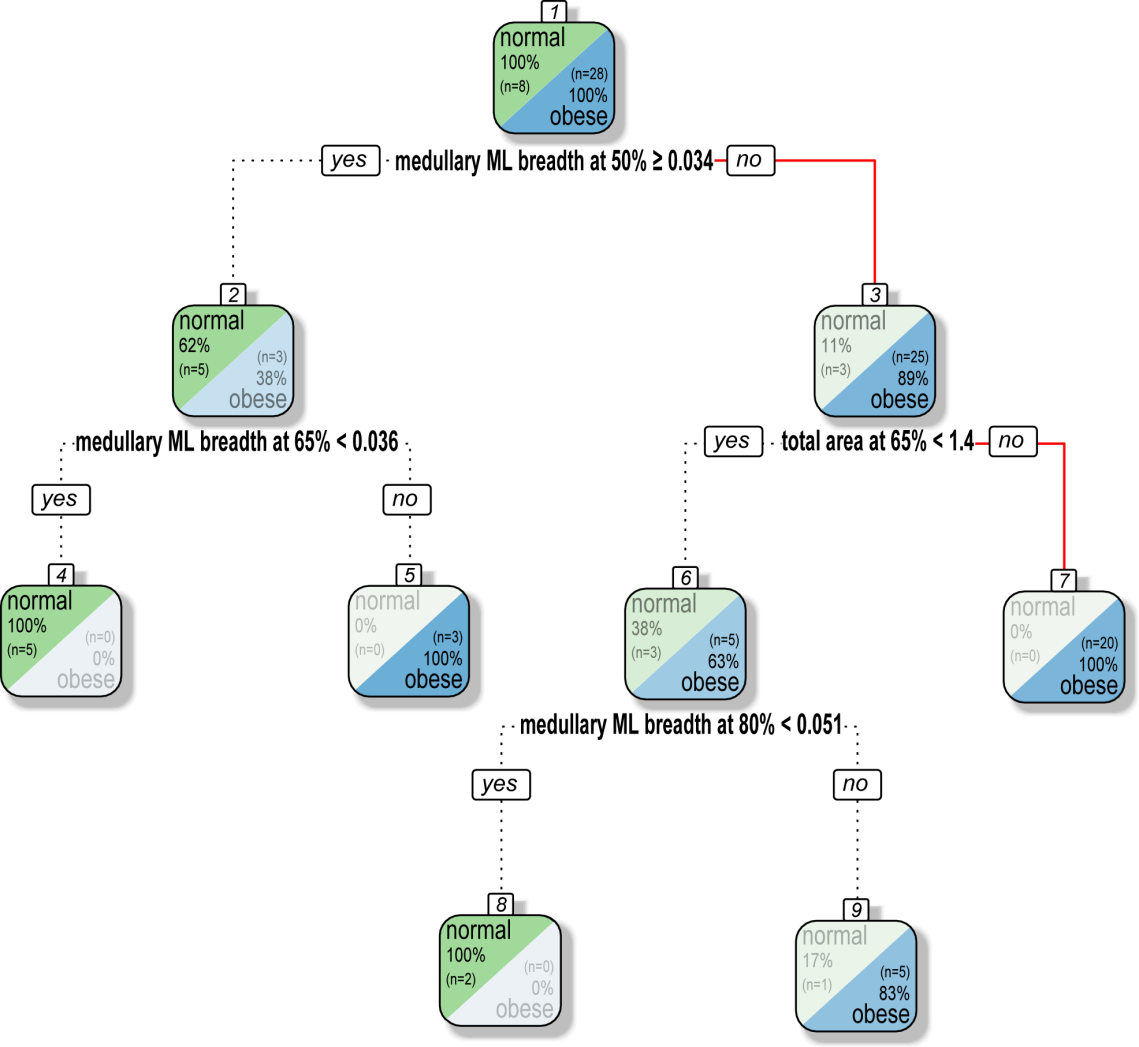
Decision tree to distinguish normal and obese categories based on body fat (BF)% data. Tycho Brahe’s classification path is identified by a plain red line. All data are adjusted according to maximum femoral length.

The decision tree generated from a Danish sample of 36 males determined that the most discriminant variables for classifying the whole sample between normal and obese categories were adjusted medullary ML breadths at 50%, 65%, and 80% of the maximum femoral length (MFL), and adjusted total area at 65% of the MFL (Fig 7; S4 and S5 Tables). The tree distinguishes almost perfectly the entire sample between normal or obese categories: 89% of the obese males have an adjusted medullary ML diameter at 50% of MFL that is smaller than 0.034 (leaf #3). When this is associated with a total area larger than 1.4, all individuals with obesity are distinguished from those with a normal BF% (leaf #7). Among individuals with an adjusted medullary ML diameter at 50% of MFL larger than 0.034 (leaf #2), those who have an associated medullary ML diameter at 65% larger than 0.036 are obese (leaf #5).

Tycho Brahe has a ML diameter at MFL 50% of 0.031 with an associated adjusted total area of 1.5 on the slice located at 65% of MFL. He therefore falls into the leaves 3 and 7 (Fig 7) and is classified as obese with 100% reliability according to the data of the tested sample. Although the small sample size of the reference sample must be acknowledged (n = 36), the results strongly support the hypothesis that Brahe was overweight in the last years of his life.

## Discussion

Our contribution provides conclusive evidence that Tycho Brahe suffered from DISH, a systemic disorder characterized by bone proliferation at spinal and extraspinal sites of the ligament and tendon attachments to bone. Moreover, isotope analysis of his skeletal remains shows that the famous astronomer had a rich diet with high protein intake. His physical status estimation suggests that he also suffered from obesity. Hereafter, we review the risk factors associated with DISH and discuss its relationship with lifestyle and its possible impact on his physical condition, health, and premature death.

### Epidemiology and biosocial determinants of DISH

Diffuse idiopathic skeletal hyperostosis, formerly referred to as Forestier’s disease, was first comprehensively described by Forestier and Rotes-Querol in 1950 [90]. Since then, the condition has been the focus of many epidemiological studies, which revealed its distribution among various contemporary populations around the world. Regardless of the population considered, the disease has been shown to strongly correlate with age: it is an unusual finding in individuals under 40 years, and its prevalence increases markedly with advancing age [20,21,91]. It is also well recognized that DISH is more common in men, with a male/female ratio ranging from 1.5:1 to 2:1 in most clinical studies [91–94]. The prevalence of the condition also shows significant ethnic and geographic variation. In the over 50 years, it has been shown to range from 2.9% in the Korean population [95] to over 10% in some Caucasian populations (e.g. [94,96]). Higher prevalence rates have occasionally been reported, for example for two large Midwest populations of the United States where 25% of males and 15% of females over 50 years had DISH, and up to 35% of males and 26% of females over 70 years [97]. In the Old World, DISH is considered by some scholars to be more frequent in Northern European people [98]. For example, the condition has been reported to be common in the Finnish population, with 3.8% of males and 2.6% of females over 40 years being affected, and as many as 10.1% of males and 6.7% of females over 70 years [91]. It is noteworthy, however, that the prevalence of DISH is also high in some populations from central Europe, such as in Hungary, where prevalence rates of 27.3% and 12.8% have been reported for males and females over 50 years, respectively [96].

The prevalence of DISH in past populations is not as well ascertained as in modern times, and it has probably been underestimated during the last decades due to misdiagnosis of many cases as ankylosing spondylitis [99]. Since the pioneering studies of Rogers and colleagues in the 1980s [68,99,100], the disease has increasingly been recognized in paleopathology. Previous studies demonstrate that DISH has been present across the world for centuries, with ancient cases reported in Europe [101–103], Africa [36], Asia [104,105], the Americas [106,107] and Australia [108]. Such research also revealed that DISH is by no means a recent condition. Skeletal changes evocative of the condition have been reported in some Neanderthal remains from the Middle Paleolithic [109,110], in skeletons from the Neolithic period [111], and in mummies from ancient Egypt [112].

Europe is currently the geographic area in which the most is known on DISH in former times, notably during the medieval and post-medieval periods. Interestingly, the disease prevalence appears to vary markedly between social groups. The condition has been repeatedly reported to be more common in monastic contexts [26, 113–116]. This has been linked to the particular lifestyle of the monks [26], although it may also, in part, be due to the overrepresentation of older adult males in skeletal sample derived from such cemeteries [25]. DISH also appears to be more frequent in individuals of high social status [25,103,117,118]. As an example, the skeletons of Grand Dukes Cosimo I (1519–1574) and his son Ferdinand I (1549–1609) both show the typical features of the disease [103]. Johann Sporck (1595–1679), another high-ranking figure known to have acted as a General during the Thirty Years’ War, also had the condition [119]. DISH was also reported, amongst other examples, in two 17^th^ century Finnish aristocrats [117], and in four royal Egyptian mummies of the 18^th^–20^th^ dynasties (Amenhotep III, Ramesses II, his son Merenptah, and Ramesses III) [112].

It has been postulated that the reasons for the association between DISH and high social status lie in lifestyle differences, and particularly in an over-rich diet in high-ranking individuals [25,26]. This hypothesis has previously been supported by the results of stable isotopic analyses, which revealed high δ^15^N values in individuals diagnosed with DISH, thus demonstrating a diet rich in animal proteins [84,120,121]. Furthermore, written and artistic sources depict some famous DISH sufferers as obese individuals, for example the Grand Dukes of Florence [103] and the General Johann Sporck [119]. The current study provides confirmatory evidence of the influence of social status and nutritional habits on the onset and manifestation of this pathology. Indeed, Tycho Brahe was a person of high extraction who has been part of the elite throughout his lifetime. Furthermore, historical sources report that he became corpulent in the last years of his life [2,122]. Obesity is confirmed by our estimation of physical status based on femoral shaft dimensions. It is also obvious from the results of our dietary study that his diet had a prestigious character, with a very high input of animal protein and/or aquatic resources. Our results thus support the view that DISH is a significant marker of lifestyle.

### DISH in a clinical context

At present, the exact etiology of DISH is still uncertain, but is most likely multifactorial [123]. Various causative agents have been suggested, including dietary, toxic, anatomic, environmental, endocrinologic, and metabolic factors [124–126]. A familial or genetic predisposition for the disease has also been proposed [127,128] but remains unproven.

DISH is reported to have life-threatening comorbidities, such as severe atherosclerotic cardiovascular disease and abdominal aortic calcification [129,130]. People with the condition have a significantly higher likelihood of being affected by coronary heart disease than non-DISH patients [131], and uncompensated or paroxysmal atrial fibrillation is more often encountered on hospital admission of patients with the condition [132]. More generally, various studies have reported that DISH carries a risk for stroke and cerebrovascular disease [133].

Besides cardiovascular comorbidities, DISH is associated with various metabolic comorbidities, including obesity, diabetes mellitus, gout, hypertension, hyperinsulinemia, dyslipidemia, and hyperuricemia [31,133]. These conditions extend beyond their simultaneous existence with DISH, but appear to be directly associated with one another. In an extensive review of clinical data, Pillai and Littlejohn [31] identified a trend in comorbidity of DISH, obesity, and adult onset (type 2) diabetes mellitus with disturbance in metabolism of growth hormone and related hormones, insulin and related hormones, and adipokines (i.e. cytokines secreted by adipose tissue). Adipokines are increased in obese individuals, and leptin (one of the adipokines) levels have been shown to promote an increase in cortical but not cancellous bone [134]. This is also supported by our results on physical status estimation based on cortical bone dimensions of the femoral diaphysis. Fat mass also correlates with the secretion of hormones from pancreatic beta cells and from adipocytes involved in reduction of bone resorption and/or osteoblast proliferation (for a review, see [135]). Owing to these metabolic derangements, patients with DISH have an increased likelihood of being affected by metabolic syndrome, and an increased risk for the development of coronary artery disease and stroke [19].

Of the various possible comorbidities, the association of DISH with obesity and late onset (type 2) diabetes are probably the better ascertained. It has been repeatedly reported in various clinical studies [20,91,92,96,126,136,137], although some others failed to confirm such a relationship [138,139]. In Tycho Brahe, the association of DISH with the first one of these conditions is evidenced by our results on physical status estimation. Whether or not he also suffered from diabetes mellitus cannot be determined with certainty, as this disease does not cause pathognomonic signs in the skeleton [140]. Although some authors tentatively diagnosed the condition on the basis of skeletal lesions [141,142], a recent evaluation of 15 supposed musculoskeletal manifestations of diabetes mellitus (e.g. DISH, osteoarthritis, osteoporosis, gout, periodontal disease) in the William M. Bass Donated Skeletal Collection revealed that no single musculoskeletal variable can be used to predict the disease [143]. Brahe’s skeleton exhibits some of these musculoskeletal manifestations, notably osteoarthritic changes and severe periodontal disease in the maxilla (for a detailed study of oral health status, see [144]). Other bone changes that can arise from diabetes, such as distal periostitis in foot phalanges, were absent, and there is no historical evidence that the astronomer suffered from secondary symptoms such as bad eyesight or gastric ulcers. That Brahe suffered from diabetes mellitus thus cannot be ascertained, but should still be viewed as a plausible hypothesis, given his dietary habits and the known high prevalence of DISH in type 2 diabetes patients [145,146].

### Possible consequences on the health status and death of Tycho Brahe

DISH is generally an asymptomatic condition, with most affected individuals not even aware of its presence [19]. As a consequence, it has been referred to by some clinicians as a ‘state’ rather than a ‘disease’ [147]. Some patients may present back and/or neck pain, restriction of mobility of the spine, and peripheral joint stiffness [19,148]. The exact role of the condition in such clinical symptoms remains, however, a matter of controversy, as some studies conclude that the degree of pain and disability in patients with DISH is higher than in healthy individuals [21,92] whereas others have failed to confirm such an association [149]. In extreme cases, the abundance of new bone formation might be responsible for stiffening of the spine to the extent that patients develop postural abnormalities [150]; this can result in compression of the spinal cord or other structures, causing severe complications [151]. Such symptoms can be excluded in Brahe’s case, considering the moderate spinal involvement he suffered. Rare complications resulting from large protruding cervical enthesophytes, such as myelopathy, dysphagia, aspiration pneumonia, esophageal obstruction, or laryngeal edema with severe dyspnea [151] can also be excluded as the axial changes on his skeleton are restricted to the thoracolumbar spine.

Whereas it is most likely that DISH represented only a minor inconvenience for Tycho Brahe and did not have any relevant impact on his daily life and health status, some comorbidities of the condition, if present, could have been life-threatening and may have played some role in the sudden illness he suffered at the end of his life. To discuss such a hypothesis, we must return to the recordings of his very last days, as described by his contemporaries. According to these textual accounts, the astronomer had two symptoms: the first was loss of consciousness (or coma) and the second was urinary retention.

What Brahe’s contemporaries perceived or described as a coma has to be understood in the context of the 17^th^ century. The analysis by Koehler and Wijdiock [152] of medical textbooks published between 1640 and 1960 provides valuable information on the subject. According to these authors “the term ‘coma’, from the Greek koma, meaning deep sleep, had already been used in the Hippocratic corpus (*Epidemica*) and later by Galen (second century AD). Subsequently, it was hardly used in the known literature up to the middle of the 17^th^ century. The term is found again in Thomas Willis’ (1621–75) influential *De anima brutorum* (1672), where lethargy (pathological sleep, which he localized in the outer cortex), ‘coma’ (heavy sleeping), *carus* (deprivation of the senses) and apoplexy (into which *carus* could turn and which he localized in the white matter) are mentioned, the sequence indicating increasingly deeper forms of unresponsiveness” [152]. They also note: “Thomas Sydenham (1624–89) mentioned the term ‘coma’ in several cases of fever” [152]. Apoplexy and stroke are amongst the causes of ‘coma’ most often encountered in this study. Although it is partly due to the methodology employed (i.e. systematic study of subject indexes and tables of contents, but also of chapters on apoplexy) this result suggests that symptoms of stroke were frequently referred to as ‘coma’ in 17^th^ century medical treatises. In this view, it is noteworthy that DISH is associated with an elevated risk of stroke and cerebrovascular disease [133].

Acute urinary retention is defined as a sudden inability to urinate although the bladder is full [153]. Aside from being painful, it can lead to renal failure or bladder dysfunction if left unaddressed, and it can result in delirium in elderly patients [154]. The exact etiology of the condition is unclear and is thought to be multifactorial, resulting from a combination of mechanical (benign prostatic hypertrophy, urethral structure, clot retention) or dynamic obstruction (increased alpha-adrenergic activity, prostatic inflammation), bladder over-distension (immobility, constipation, drugs inhibiting bladder contractility, high alcohol intake), and neuropathic mechanisms (diabetic cystopathy, multiple sclerosis) [155]. In males, most cases are associated with benign prostatic hyperplasia [155–157], i.e. an enlargement of the prostate gland that occurs in almost all men as they age. In addition, diabetic neuropathy and stroke are frequent causes of acute urinary retention [153,158]. Thus, an association between DISH, diabetes, and benign prostatic hypertrophy could possibly have been the cause of Brahe’s symptoms. These pathological states may be associated with a metabolic syndrome that is very likely to have occurred, as suggested by information on his lifestyle, as well as by our findings on his diet and his physical status.

Considering diabetes mellitus as a possible comorbidity with DISH and obesity in Tycho Brahe, two specific complications of this disease must be considered as possible causes of his death, namely diabetic ketoacidosis (DKA) and the hyperosmolar hyperglycemic state (HHS). DKA is an acute and possibly life-threatening metabolic complication of diabetes resulting from severe insulin deficiency, and subsequent hyperglycemia and metabolic acidosis [159]. It occurs most commonly in autoimmune type 1 diabetes but patients with type 2 diabetes are also at risk during the catabolic stress of acute illness, such as an infection [159,160]. To this extent, it is noteworthy that urinary tract infection is a common accompanying phenomenon in patients with diabetes mellitus, and is generally more severe and carries worse outcomes in patients with type 2 diabetes [161]. Symptoms of DKA include dehydration, diffuse abdominal pain, vomiting, tachypnea, confusion, and occasionally loss of consciousness [159,162], some of which are consistent with the description of Brahe’s illness. However, DKA is generally accompanied by excess urination rather than urinary retention [159], making it a rather unlikely diagnosis.

HHS, on the other hand, is a syndrome characterized by extreme elevations in serum glucose concentrations and hyperosmolality, in the absence of significant ketoacidosis [159,163]. Contrary to DKA, it occurs more commonly in patients with type 2 diabetes [163]. Although the pathogenesis of HHS is not perfectly understood to date, the condition appears to result from synergistic factors including insulin deficiency and increased levels of counter-regulatory hormones [159]. As for DKA, the most common precipitating factor is infection [159,164]. Symptoms include signs of dehydration, weakness, legs cramps, delirium, and an altered level of consciousness that evolves not infrequently to profound lethargy or coma [159,162,164]. Although HHS typically causes increased urination, cases have also been reported where patients present with acute urinary retention associated with benign prostatic hypertrophy [165]. Thus, such a syndrome appears as a possible, although putative, cause of the symptoms Brahe endured during his very last days. This hypothesis is compatible with the known fatal outcome of his sudden illness, since mortality associated with HSS is between 10% and 20% in current clinical practice (i.e. about 10 times higher than in patients with DKA) [163].

Finally, another factor that could have conditioned the death of the astronomer, along with having an additive effect on his obesity and metabolic issues (e.g. promoting diabetes) is an excessive consumption of alcohol. In view of our knowledge of the way of life of renaissance noblemen, we can assume that Brahe consumed more alcohol than most of his contemporaries. Furthermore, historic accounts relate that he was notorious for drinking great quantities of wine and other alcoholic beverages [166,167]. Alcohol abuse may cause serious metabolic disturbances, among which alcoholic ketoacidosis, a condition due to the accumulation of β-hydroxybutyrate and acetoacetic acid in the body [168]. As in DKA, symptoms may include abdominal pain, vomiting, tachypnea, and occasionally altered consciousness [169]. This condition manifests itself suddenly, and constitutes in modern days a fairly common cause of sudden, unexpected death in heavy drinkers [170,171]. The so-called “ketoalcoholic death” [168] thus appears as a plausible, albeit hypothetic, diagnosis in Tycho Brahe.

## Conclusions

Previous studies of skeletal and hair remains of Tycho Brahe have ruled out the possibility that he died from a violent death (chronic poisoning) and excluded chronic long-term kidney disease and renal osteodystrophy, nutritional osteomalacia, vitamin D and calcium deficiency from gastrointestinal malabsorption syndromes, osteopenia, or osteoporosis [5]. The current paleopathological study provides further evidence about his health status, by revealing that he suffered from both diffuse idiopathic skeletal hyperostosis and obesity. These results, along with those of the isotopic analysis, give a glimpse into the lifestyle of the famous astronomer, revealing the dietary excesses a 16^th^ century high-ranking individual could have afforded (high caloric intake and presumably excessive alcohol consumption). They also reveal the possible health consequences such a prestigious way of life could have had. Although this study does not allow a definite diagnosis to be reached, it highlights plausible reasons for the sudden illness and premature death of the famous astronomer, notably conditions resulting from so-called civilization diseases, which occur with high frequency in DISH patients.

## Supporting information

S1 Table Grades of degenerative changes of the spine (spondylosis and spondyloarthrosis) in Tycho Brahe's skeleton (according to classification systems of Vyhnánek, Stloukal 1971 and Üstündağ 2009 [15,16]). (XLSX)

S2 Table Grades of osteoarthritic changes in the appendicular skeleton of Tycho Brahe (according to classification system of Stloukal, Vyhnánek 1975 [14]). (XLSX)

S3 Table Stages of entheseal changes in main muscle attachment sites of Tycho Brahe (according to classification system of Villotte 2006 [13]). (XLSX)

S4 Table The values of femoral dimensions (maximum femoral length, medio-lateral endosteal breadth) of the reference sample used in the decision tree for physical status estimation of Tycho Brahe. (XLSX)

S5 Table The statistic values of femoral dimensions of the reference sample and Brahe's values used in the decision tree for his physical status estimation. (XLSX)
